# Where to Start? Alternate Protein Translation Mechanism Creates Unanticipated Antigens

**DOI:** 10.1371/journal.pbio.0020397

**Published:** 2004-10-26

In the spirit of good health, cells are constantly subjecting their protein contents to immunological surveillance by cytotoxic (killer) T cells. Tens of thousands of major histocompatibility complex (MHC) class I molecules cradle peptides (bits of proteins) on cell surfaces, and T cells detect any suspicious peptides with extreme sensitivity. If a cell is infected with a virus, peptides created from viral DNA will end up on the cell's surface as antigens, triggering immunological red flags.

Most—but not all—of the peptides presented by MHC class I molecules are created by conventional cellular mechanisms: with the help of a ribosome, three mRNA nucleotides (a codon) are decoded into a corresponding amino acid, which is strung as the next link on an elongating peptide. Most peptides begin with the amino acid methionine, coded by the mRNA nucleotide triplet A-U-G (AUG). But some peptides are “cryptic,” arising from normally untranslated regions of mRNA or initiated with codons other than AUG.

Previous studies suggested that an unconventional translation mechanism creates some cryptic peptides. But how? And why? Only one type of translation initiator, transfer RNA (tRNA), specific for AUG and loaded with a methionine molecule, is known. Protein synthesis beginning at alternate codons has been attributed to imprecise pairing between the methionine translation initiator and mRNA. This, however, does not explain proteins that do *not* begin with methionine.

Only two mechanisms for building non-methionine-initiated peptides have been discovered. In a new study, Susan R. Schwab et al. characterize one of them, the CUG-initiated translation of a peptide starting with leucine instead of methionine.

The authors explored cellular translation by engineering cells to create peptides of interest and present them through matching MHC molecules on the cells' surfaces. Then, by harnessing the exquisite sensitivity of T cells to probe for antigens on MHC molecules, they could identify which peptides were created under different experimental conditions.

Their findings point to a unique translation mechanism. In the other known example of a non-methionine-initiated peptide, translation beginning at GCU or CAA is guided by a specific folded structure of mRNA nucleotides called the internal ribosome entry site. Schwab et al. have found that no similar structure is necessary for CUG-initiated translation. However, similar to the standard mechanism of AUG initiation, they found that ribosomes do scan for CUG. Additionally, the presence of a specific ribosome-binding sequence in mRNA (the “Kozak context”) near a CUG site can enhance the efficiency of initiation there.

Schwab et al. have also suggested a possible purpose for this translation mechanism. Under stress, cells can down-regulate conventional translation, which curbs the production of viral proteins in the event of an infection but also suppresses the creation of antigens needed to flag down T cells for an immune response. Here, Schwab et al. report that peptides starting with leucine were produced in the absence of the protein eIF2, which normally aids in AUG-initiated peptide synthesis. Cells under stress slow conventional translation by restraining the function of eIF2. Therefore, CUG-initiated translation, which works without eIF2, might provide an out for stressed cells needing to create peptides. This alternative could be a great way to avoid pumping out viral proteins and still create antigens for T cell surveillance—unless, of course, viruses take advantage of the loophole for their own peptide production.

